# Comparative Evaluation of Machine Learning Models for Subtyping Triple-Negative Breast Cancer: A Deep Learning-Based Multi-Omics Data Integration Approach

**DOI:** 10.7150/jca.93215

**Published:** 2024-05-30

**Authors:** Shufang Yang, Zihui Wang, Changfu Wang, Changbo Li, Binjie Wang

**Affiliations:** Department of Imaging, Huaihe Hospital of Henan University, Kaifeng 475000, P. R. China.

**Keywords:** Artificial Intelligence, Deep Learning, Magnetic Resonance Imaging, Triple-Negative Breast Cancer, Multi-Omics, Prediction Model, Multi-Omics Analysis, Bayesian Optimization

## Abstract

**Objective:** Triple-negative breast cancer (TNBC) poses significant diagnostic challenges due to its aggressive nature. This research develops an innovative deep learning (DL) model based on the latest multi-omics data to enhance the accuracy of TNBC subtype and prognosis prediction. The study focuses on addressing the constraints of prior studies by showcasing a model with substantial advancements in data integration, statistical performance, and algorithmic optimization.

**Methods:** Breast cancer-related molecular characteristic data, including mRNA, miRNA, gene mutations, DNA methylation, and magnetic resonance imaging (MRI) images, were retrieved from the TCGA and TCIA databases. This study not only compared single-omics with multi-omics machine learning models but also applied Bayesian optimization to innovatively optimize the neural network structure of a DL model for multi-omics data.

**Results:** The DL model for multi-omics data significantly outperformed single-omics models in subtype prediction, achieving a 98.0% accuracy in cross-validation, 97.0% in the validation set, and 91.0% in an external test set. Additionally, the MRI radiomics model showed promising performance, especially with the training set; however, a decrease in performance during transfer testing underscored the advantages of the DL model for multi-omics data in data consistency and digital processing.

**Conclusion:** Our multi-omics DL model presents notable innovations in statistical performance and transfer learning capability, bearing significant clinical relevance for TNBC classification and prognosis prediction. While the MRI radiomics model proved effective, it requires further optimization for cross-dataset application to enhance accuracy and consistency. Our findings offer new insights into improving TNBC classification and prognosis through multi-omics data and DL algorithms.

## Introduction

Breast cancer is one of the most common malignant tumors in women, and triple-negative breast cancer (TNBC) is one of the most invasive and aggressive subtypes of breast cancer 1. TNBC lacks hormone receptors and HER2 overexpression, making it currently lacking effective targeted therapies 2-4. Accurate classification prediction is crucial for guiding personalized treatment and prognosis assessment in patients 5-7. However, currently, there is a lack of a unified and reliable classification method, and the diagnostic methods based on traditional clinical pathological characteristics and molecular markers also have certain limitations 8-10. Therefore, it is important to conduct research to seek new and reliable predictive models.

Genetic and epigenetic features as potential indicators for classification prediction are significant in breast cancer as they contribute to the diagnosis and selection of treatment strategies, including gene mutations, DNA methylation, mRNA expression, and miRNA expression 11-13. Each molecular feature can provide richer biological information and contribute to breast cancer classification prediction. However, the accuracy and robustness of classification prediction solely using these molecular features remain limited. Approaching from the perspective of integrating multi-omics data can cover more levels of information, thereby improving the accuracy and reliability of predictive models 14. Therefore, this study utilized various molecular feature data of TNBC subtyping from public databases such as TCGA and TCIA, including mRNA, miRNA, gene mutations, and DNA methylation, as well as magnetic resonance imaging (MRI) radiomics data, to construct a more comprehensive and accurate classification prediction model.

Deep learning (DL), as the latest generation of artificial intelligence technology, has made significant progress in areas such as image and speech recognition, natural language processing, etc. 15-17. Similarly, DL has also shown great potential in the medical field 18-20. In this study, we applied DL techniques to establish predictive models to further improve TNBC classification prediction accuracy. Specifically, we adopted DL models for multi-omics data and MRI radiomics DL models and optimized the neural network structure using Bayesian optimization methods 21, 22. The integration of these models may enhance our understanding of TNBC subtyping classification and provide more reliable evidence for clinical decisions.

The primary goal of this investigation was to assess the effectiveness of various machine learning and DL approaches in predicting the classification of TNBC. We aimed to enhance the precision and reliability of these predictions by leveraging multi-omics data alongside advanced DL techniques. In particular, the study compared the efficacy of single-omics machine learning models against more sophisticated DL models for multi-omics data and MRI radiomics DL models. The evaluation of these models was thorough, utilizing cross-validation and transfer testing methodologies. Key metrics employed for assessment included accuracy, precision, recall, F1 score, and the area under the receiver operating characteristic (ROC) curve (AUC), providing a comprehensive analysis of each model's strengths and limitations. Through this research, we anticipated developing more accurate and dependable tools for classifying and diagnosing TNBC subtyping, thereby facilitating personalized treatment options for patients and enhancing the accuracy and robustness of prognosis assessment and clinical decision-making specific to TNBC cases.

**Figure 1:**
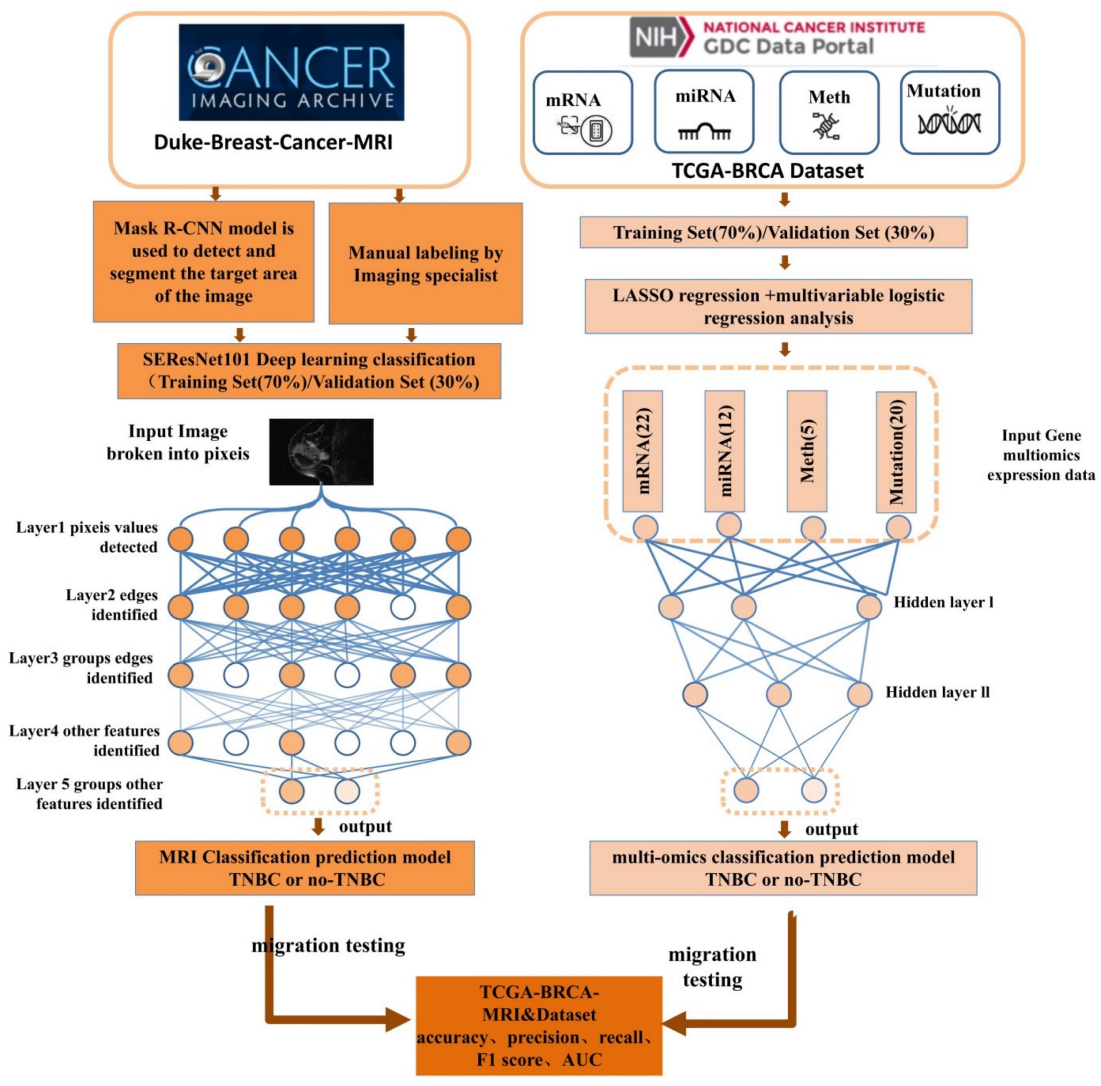
Construction process of the DL prediction model.

TNBC is a notably malignant and invasive subtype of breast cancer, making accurate and reliable classification a crucial aspect of clinical management. This study endeavored to refine TNBC classification accuracy and dependability by exploiting multi-omics data and implementing DL methodologies. By integrating a broad spectrum of molecular features and employing cutting-edge DL technologies, we developed predictive models, notably DL models for multi-omics data and MRI radiomics DL models (Figure 1). Through meticulous performance evaluation and model comparison, this research aimed to furnish clinicians with more effective tools for TNBC classification, thereby enabling personalized patient treatment strategies and improving the precision and stability of prognosis assessments.

## Materials and Methods

### TCGA database utilization and data compilation

Data were retrieved from the TCGA database (https://cancergenome.nih.gov/), which facilitated the download of the TCGA-BRCA dataset (The Cancer Genome Atlas, TCGA; Breast Invasive Carcinoma, BRCA). The dataset encompassed various data types, including 1,089 cases of copy number variation (CNV) data, 977 cases of mutation data, 1,097 cases of methylation data, 1078 cases of miRNA expression data, 1093 cases of mRNA expression data, and 137 samples from the TCGA-BRCA-MRI dataset. Relevant data were downloaded based on sample IDs from the TCIA (The Cancer Imaging Archive) database and were supplemented with clinical information (Supplemental File 1-2). The classification of samples as TNBC was determined based on the negative status of Estrogen Receptor (ER), Progesterone Receptor (PR), and Human Epidermal Growth Factor Receptor 2 (HER2). Given the public nature of these databases, ethical approval or informed consent was not requisite for the utilization of this data.

### Transcriptomic features extraction and classification prediction model construction

Differentially expressed genes (DEGs) were selected using the R language limma package, with |logFC| > 2 and P < 0.05 as filtering criteria. The MAD (Median Absolute Deviation) for each gene was calculated based on the differential gene expression profiles, and the bottom 50% of genes with the smallest MAD were removed. The most significant DEGs (|logFC| > 4 and P < 0.005) were selected for LASSO regression model construction. A multivariable logistic regression model was built to classify TCGA-BRCA samples based on the selected genes, using the λ value for gene selection 23.

### Mutation features extraction and classification prediction model construction

Genes with high frequencies of mutation events were selected. Then, a LASSO regression model was employed to choose genes, and a subgroup composed of multiple feature genes was created. A multivariable logistic regression model was subsequently constructed to classify TCGA-BRCA samples, and the AUC value was computed 24.

### MRI image dataset

The study participants were selected from the Duke-Breast-Cancer-MRI and TCGA-BRCA-MRI datasets available in the public Cancer Imaging Archive (TCIA) provided by the National Cancer Institute (NCI) in the United States. The following criteria were excluded: (1) preoperative interventions, such as neoadjuvant therapy or tumor resection that affected the tumor morphology prior to the MRI examination; (2) multiple malignant lesions, which could affect the accuracy of the binary classification due to the presence of multiple image features in cases of multiple breast cancers; (3) incomplete clinical or pathological information. Finally, 874 samples from Duke-Breast-Cancer-MRI and 84 samples from TCGA-BRCA were retained. The samples from Duke-Breast-Cancer-MRI were randomly divided into a training set and a validation set in a 7:3 ratio, while the TCGA-BRCA images were used as an external test set. Two experienced radiologists, with 6 and 4 years of experience, respectively, confirmed the index lesions in each MRI image. Multiple views of the indexed tumor images were captured, including longitudinal and transverse sections. Tumor size was measured in the longitudinal section. All imaging data and clinical data were obtained from public databases. Therefore, ethical approval or informed consent was not required 25.

### MRI Image model dataset processing

Like most other studies using such models in the medical field, we trained our model on the Duke-Breast-Cancer-MRI dataset. To fine-tune the model, we used a training and validation setting. The model was trained to learn image patterns for each class on the training set (70%), while the validation set (30%) was initially not visible during training and, therefore, excluded from the samples. Data were partitioned in the sample space rather than the image space, so different images of the same sample were placed in one of these two groups and appeared in only one group. To demonstrate the transferability of the model, we tested it on the "TCGA-BRCA" dataset from TCIA 26.

### MRI image preprocessing

The MRI head image dataset was collected, and each original MRI image was divided into overlapping local blocks with a size of 32×32 pixels. The adaptive histogram equalization algorithm (CLAHE method) was applied to each local block with the following parameters: contrast limit (clipLimit) set to 2.0 and grid size (tileGridSize) set to 8×8. The locally equalized image blocks were further fused using the mean fusion method. The fused image was then standardized by scaling the pixel values to the range of [0-1], achieved by dividing each pixel value by 255. This approach ensured uniform model input sizing and mitigates the noise present in the external image, addressing the issue of intensity heterogeneity in MRI to ensure the correct learning of head image segmentation information by the model 27, resulting in improved robustness and accuracy of the Mask R-CNN model 28.

### Object detection and segmentation of image regions using the Mask R-CNN model

To ensure consistency of the input data, a histogram equalization algorithm was applied, followed by resizing the images to a fixed size of 256×256 pixels and performing grayscale normalization. Tumor regions in MRI images were manually annotated by expert radiologists using OsiriX software. Each annotation for an image should include bounding boxes and pixel-level segmentation masks to support object detection and pixel-level segmentation tasks. The Mask R-CNN model was constructed and trained using the PyTorch DL framework. The stochastic gradient descent (SGD) optimization function was employed during training, and the model's weights were adjusted to accurately detect and segment tumor regions. Pretrained weights from the Mask R-CNN model were used as initial weights and fine-tuned on the "Duke-Breast-Cancer-MRI" dataset. The network architecture was modified to accommodate MRI images by changing the input channel size, and hyperparameters such as learning rate, batch size, and iteration count were specified. The performance of the trained model was evaluated on a validation set, measuring metrics, such as accuracy, recall, F1 score for object detection, and Dice coefficient for pixel-level segmentation. Cross-validation was performed by splitting the dataset into non-overlapping folds to provide an average evaluation of the results. The trained model was then used to detect, classify, and segment targets in newly unseen MRI images, and the results were quantitatively evaluated by comparing them with manual annotations. Analysis of error types and common mistakes was conducted, and adjustments and improvements were made accordingly based on specific situations 29.

### Training, validation, and transfer learning of an MRI image binary classification model using SE-ResNet101

SE-ResNet101 is a deep convolutional neural network model commonly used for image classification tasks. For binary classification, its output is typically passed through a fully connected layer and applies a softmax or sigmoid activation function to obtain classification probabilities. The formula for SE-ResNet101 in a binary classification task is as follows:

Feature Extraction: SE-ResNet101 consisted of convolutional layers, batch normalization layers, and activation functions to extract features from input images. This part could be represented as follows:



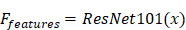




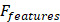
 represented the extracted features from the input image. The Squeeze-and-Excitation (SE) module was used to learn the importance of each channel's feature. It consisted of two operations: Squeeze and Excitation. In the Squeeze operation, the feature map was compressed into a global representation using global average pooling (GAP).


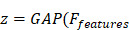
)

Among them, z represented the global description.

The excitation operation employed a small, fully connected neural network to learn the weights for each channel in order to capture the significance of features within the channel. This neural network typically consisted of one or more fully connected layers. Assuming that the output of this neural network was a vector, the excitation operation could be represented as:



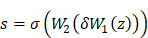



In this context, the activation function, commonly ReLU or sigmoid, represented Dropout or other regularization operations. Additionally, they were the weight matrices of two separate and fully connected layers. Feature reweighting entailed utilizing the learned weight vector to reweight the original feature map, denoted as 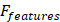:


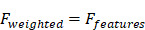
⊙s

In this regard, the symbol ⊙ is used to denote element-wise multiplication.

Classification Section: The feature map after heavy re-weighting processing was passed to a fully connected layer, and then softmax or sigmoid activation function was applied to obtain the output probability for the binary classification task:



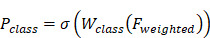



Among them, 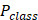 is the output probability for binary classification tasks, 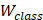 is the weight matrix used for classification, and 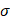 is the sigmoid activation function.

**Figure 2:**
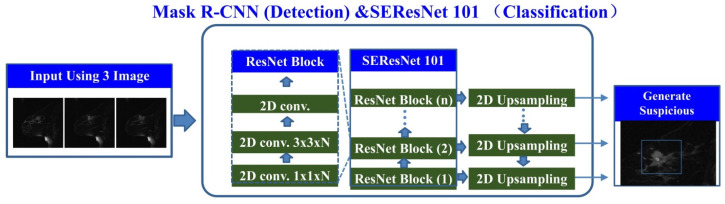
Constituents of SEResNet101 architecture.

The model was typically trained using the Cross-Entropy Loss function and optimized through gradient descent to update the weights and minimize the loss function. In the SEResNet101 architecture, the output bounding box cropping parameters for the detected abnormal regions by Mask R-CNN are fed into SEResNet101 for classification. Figure 2 illustrates the SEResNet101 model, which consists of 16 residual blocks. Each block comprises a 1×1 convolutional layer, a 3×3 convolutional layer, and another 1×1 convolutional layer. The residual connections extended from the beginning to the end of each block. The output of the final block was connected to a fully connected layer through a sigmoid function to obtain the probabilities for binary classification prediction.

Each block consisted of a 1×1 convolutional layer, a 3×3 convolutional layer, and another 1×1 convolutional layer. The bounding boxes detected by Mask R-CNN were used to crop the lesions on three images, with the output being the probability of a positive diagnosis. A probability greater than or equal to 0.5 indicated a positive result, while a probability less than 0.5 indicated a negative result 27.

### Processing of genomic multimodal data in gene omics models

Genomic data, including CNV data, mutation data, methylation data, miRNA expression data, and mRNA expression data, were selected from the TCGA database as the source of raw data for the testing set of the gene omics multimodal DL model. Samples and features with missing data exceeding 20% were filtered, and the "impute" package in R was used to further address missing values in the omics dataset. A total of 840 samples were retained for training, validation, and testing of the DL model. Among them, the testing set consisted of 137 samples from the "TCIA-BRCA-MRI" dataset, while the training and validation sets were randomly partitioned after excluding the testing set 30.

### Dimensionality reduction and DL model construction for multi-omics data

In this study, we utilize the neighborhood component analysis (NCA) for feature selection and dimensionality reduction of multi-omics data. The multi-omics data feature set is defined as X = {x1, x2, x3, ..., xn} ∈ RP, where each feature corresponds to the classification information ci for each sample. This results in an n × p matrix consisting of n samples and p feature variables. NCA achieves dimensionality reduction by constraining the quadratic distance metric to a low rank. The lower-level distance metric can be defined as follows:



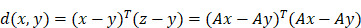



Under the objective function presented below, the inability to access testing data during training necessitates the utilization of Leave One Outperformance.



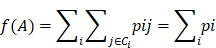



In MATLAB R2019b, a two-layer neural network model is built for a binary classification task. The target is optimized using a cross-entropy loss function. The loss function and hyperparameters are optimized through scaled conjugate gradient backpropagation and Bayesian hyperparameter optimization (Bayes-Opt). The hidden layer uses a symmetric hyperbolic tangent activation function with an initial mean of zero, while the output layer uses a sigmoid function with adjustable parameters for output. The output values of the neural network range between 0 and 1, which are used to determine the binary classification result:



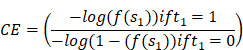



In this study, when t1 = 1, it signifies the assignment of sample C1 = Ci. The optimization of the entire network and its parameters was conducted using grid search and Bayes-opt optimization. The basic formulas are presented as follows:



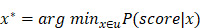



The objective function can be substituted with mean square error or cross-entropy, and the optimal surrogate function can be found utilizing a set of hyperparameters that yield the best model performance. These functions are iteratively applied to the actual objectives 31.

## Results

### Analysis of differential gene expression and diagnostic performance of models in TNBC-positive and -negative samples

We compared the mRNA expression profiles of TNBC-positive and -negative samples and found a 10.9% (*P <* 0.05) difference in gene expression between TNBC-positive and -negative samples. Among them, TNBC-positive samples had 718 upregulated genes and 444 downregulated genes (Figure S1A). To investigate the characteristics of these DEGs further, we conducted GO and KEGG pathway enrichment analysis. The GO enrichment analysis results showed that DEGs in TNBC-positive samples were mainly enriched in hormone regulation and immune cell-related pathways (Figure S1B). Additionally, through KEGG enrichment analysis, we found that DEGs in TNBC-positive samples were enriched in cytokine-related pathways (Figure S1C).

**Figure 3:**
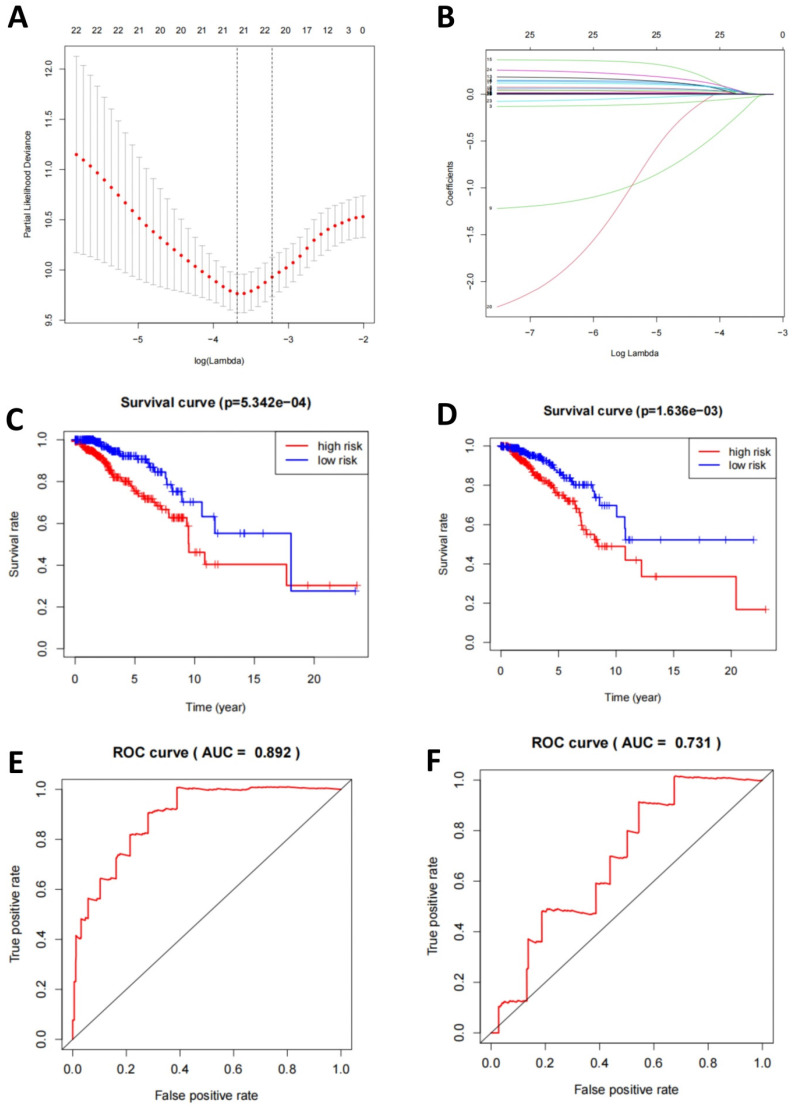
** TNBC classification prediction based on mRNA-seq data features.** Note: (A) Regularization path plots demonstrate the changes in model coefficients at different λ values for LASSO regression. The optimal λ value is determined through cross-validation and indicated by a vertical dashed line in the plot. (B) Model coefficients vary with λ, which helps in selecting the appropriate λ value for model sparsity. (C-D) Survival curves for the training and validation sets compare the survival time of high-risk and low-risk groups. The p-value indicates statistically significant differences between the two groups. (E-F) ROC curves for the training and validation sets illustrate the relationship between the true positive rate and the false positive rate of the model predictions. Additionally, the AUC value is provided to evaluate the diagnostic ability of the model.

At the transcriptomic level, we selected DEGs for LASSO regression and used the LASSO results to select 22 genes for multivariate logistic regression machine learning (Figure 3A-B). Subsequently, we plotted the survival curves and compared the survival times between the high-risk and low-risk groups. Compared to the validation set, the model demonstrated more significant differences in the training set (Figure 3C-D). Finally, the ROC curve displayed the relationship between the true positive rate and the false positive rate of the model's predictions. The results showed that the AUC of the model in the training set was 0.892, while in the validation set, it was 0.731 (Figure 3E-F). This indicates that the model has good diagnostic ability.

### Characteristics of miRNA-seq in TNBC-positive samples and evaluation of a multivariate logistic regression model

**Figure 4:**
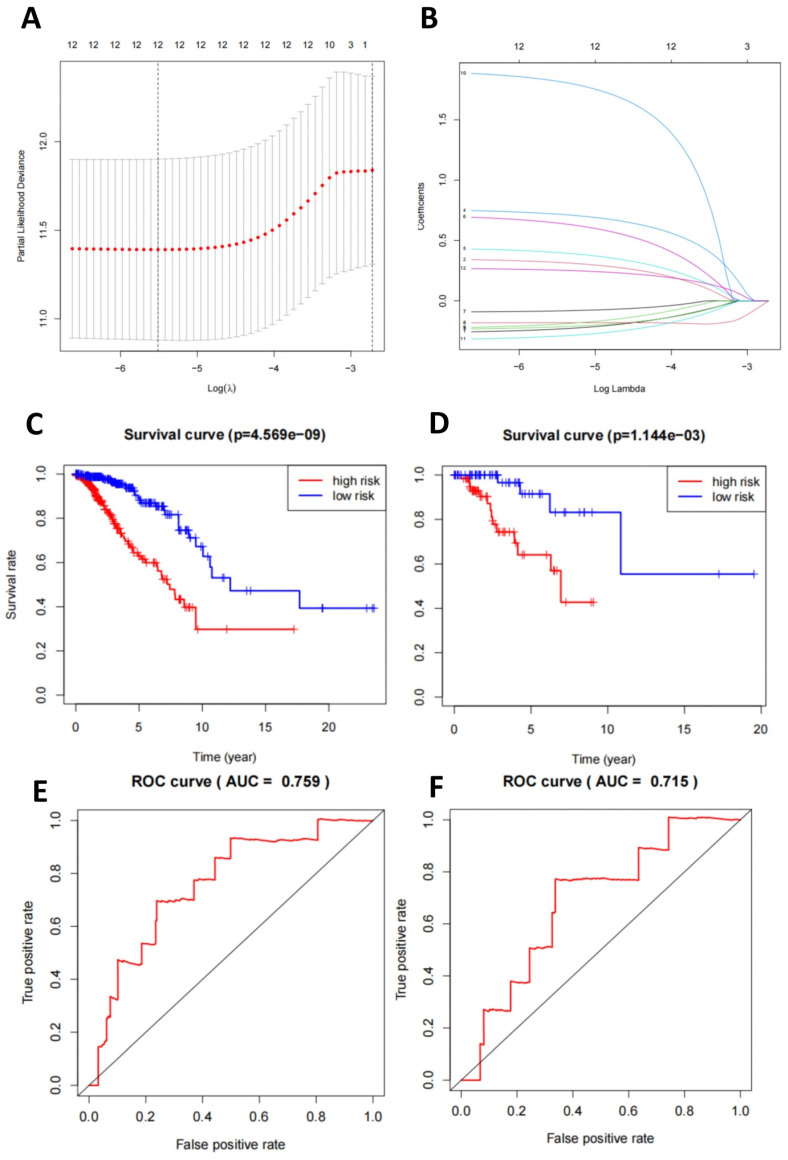
** Machine learning model for TNBC subtyping prediction based on miRNA-seq data features.** Note: (A) The regularization path plot of LASSO regression illustrates the variation of model coefficients at different λ values. The optimal λ value is determined by cross-validation and can be observed from the vertical dotted line in the plot. (B) The model coefficients are shown as λ changes to facilitate the selection of λ for model sparsification. (C-D) The survival curves of the model's training and validation sets compare the survival times between high-risk and low-risk groups, with p-values indicating statistically significant differences between the two groups. (E-F) The ROC curves of the model's training and validation sets demonstrate the relationship between the true positive rate and false positive rate, as well as the AUC value, which is used to assess the diagnostic ability of the model.

We conducted a detailed study on the characteristics of miRNA-seq in TNBC-positive and -negative samples. In the miRNA-seq in TNBC-positive samples, the expression of miR-21, miR-155, and miR-210 increased significantly (Figure S2A-D, Figure S3A-B, Figure S4A-D). Through selection, we constructed a LASSO regression model to determine DEGs, resulting in a combination of 12 miRNAs (Figure 4A-B). Based on this, we established a multivariate logistic regression model. We plotted the survival curves and compared the survival times between the high-risk and low-risk groups. Compared to the validation set, the model showed more significant differences in the training set (Figure 4C-D). Finally, we displayed the relationship between the true positive rate and false positive rate of the model's predictions through the ROC curve. The results showed that the AUC of the model in the training set was 0.759, while in the validation set, it was 0.719 (Figure 4E-F). This indicates that the model has good diagnostic ability.

### Analysis of mutation status and associated TMB mutation genes in TNBC-positive and -negative samples

**Figure 5:**
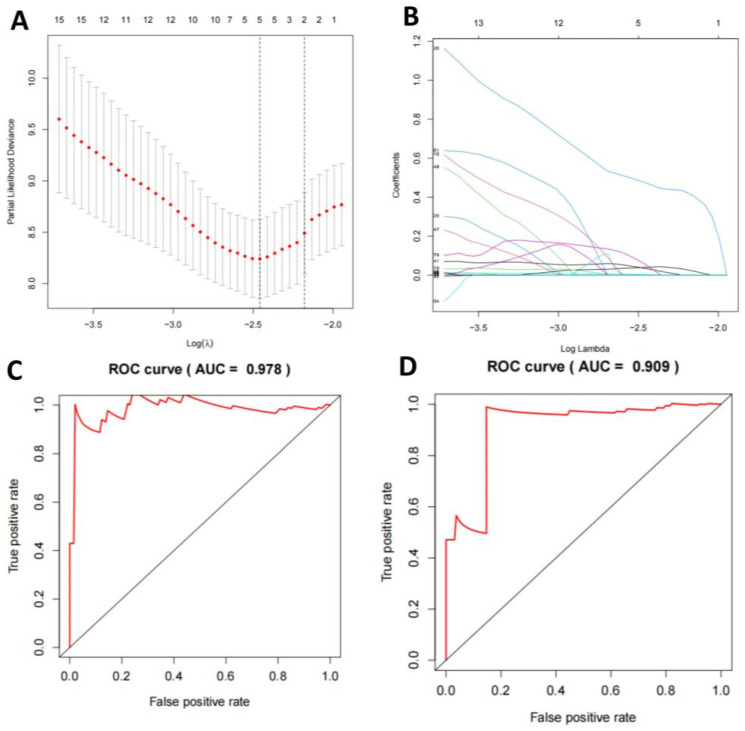
** Machine learning model for TNBC subtyping prediction based on mutation data features.** Note: (A) The regularization path plot of LASSO regression shows the variation of model coefficients at different λ values. The optimal λ value is determined through cross-validation and can be seen from the vertical dotted line in the plot. (B) The model coefficients are shown as λ changes to aid in the selection of λ for model sparsification. (C-D) The ROC curves of the model's training and validation sets display the relationship between the true positive rate and false positive rate, as well as the AUC value, which is used to evaluate the diagnostic ability of the model.

We compared the mutation status of TNBC-positive and -negative samples and found that missense mutations were the most common, mainly single nucleotide polymorphisms. Notably, the mutation frequencies of PIK3CA, TP53, TTN, CDH1, GATA3, MUC16, MAPJK1, KMT2C, HMCN1, and ALG were higher in TNBC-positive samples (Figure S5A-F). For selecting TMB mutation genes, we employed a LASSO regression model to select feature genes and identified a set consisting of 5 mutation genes (Figure 5A-B). Based on these results, we established a multivariate logistic regression model, which achieved an AUC of 0.978 in the training set and 0.909 in the validation set (Figure 5C-D).

### Analysis of DNA Methylation Features and the Multivariate Logistic Regression Model

**Figure 6:**
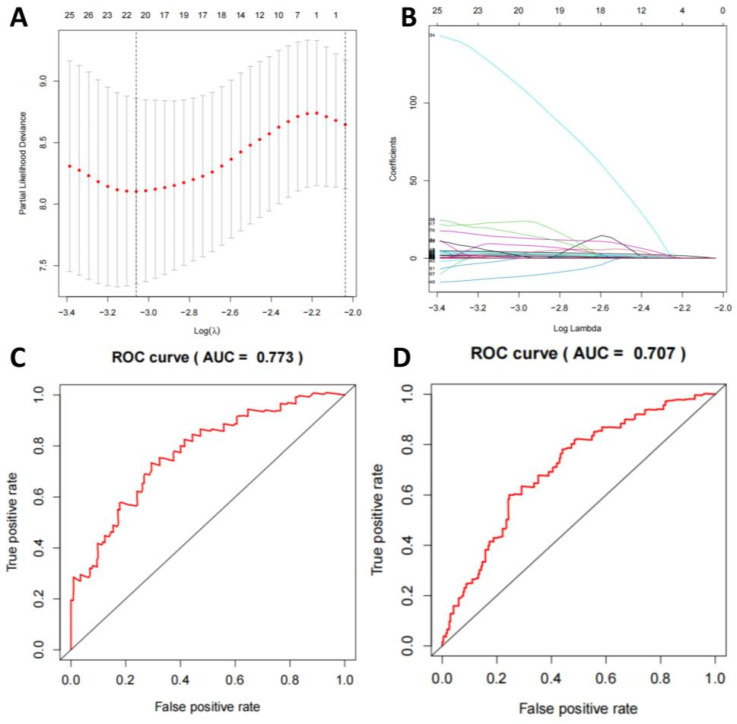
** Machine learning model for TNBC subtyping prediction based on DNA methylation features.** Note: (A) LASSO regression algorithm is employed to select the optimal DNA methylation features. (B) The AUC of the DNA methylation model in the training set. (C) The AUC of the DNA methylation model in the validation set.

We compared the features of DNA methylation in TNBC-positive and -negative samples and selected 472 genes with frequent methylation events. After using a LASSO regression model to screen the genes, we identified a combination of 20 methylation genes (Figure 6A-B). Based on this, we established a multivariate logistic regression model, which yielded an AUC of 0.773 in the training set and 0.707 in the validation set (Figure 6C-D).

### Successful classification of TNBC subtyping using a DL model based on multi-omics data

We integrated 22 mRNA, 12 miRNA, 5 gene mutations, and 20 DNA methylation predictive markers to construct a predictive model with better performance. We then used a LASSO regression model to model all predictive markers and optimized the regularization parameters and hidden layer structure of the neural network using Bayesian Optimization with 10-fold cross-validation. The final neural network model consisted of two hidden layers with 7 nodes each and had two output categories. The regularization parameter was set to 0.9999. We used a "trainscg" training function and the scaled conjugate gradient method to update the weights and biases. Using cross-entropy as the performance evaluation criterion, we built a comprehensive overall model.

**Figure 7:**
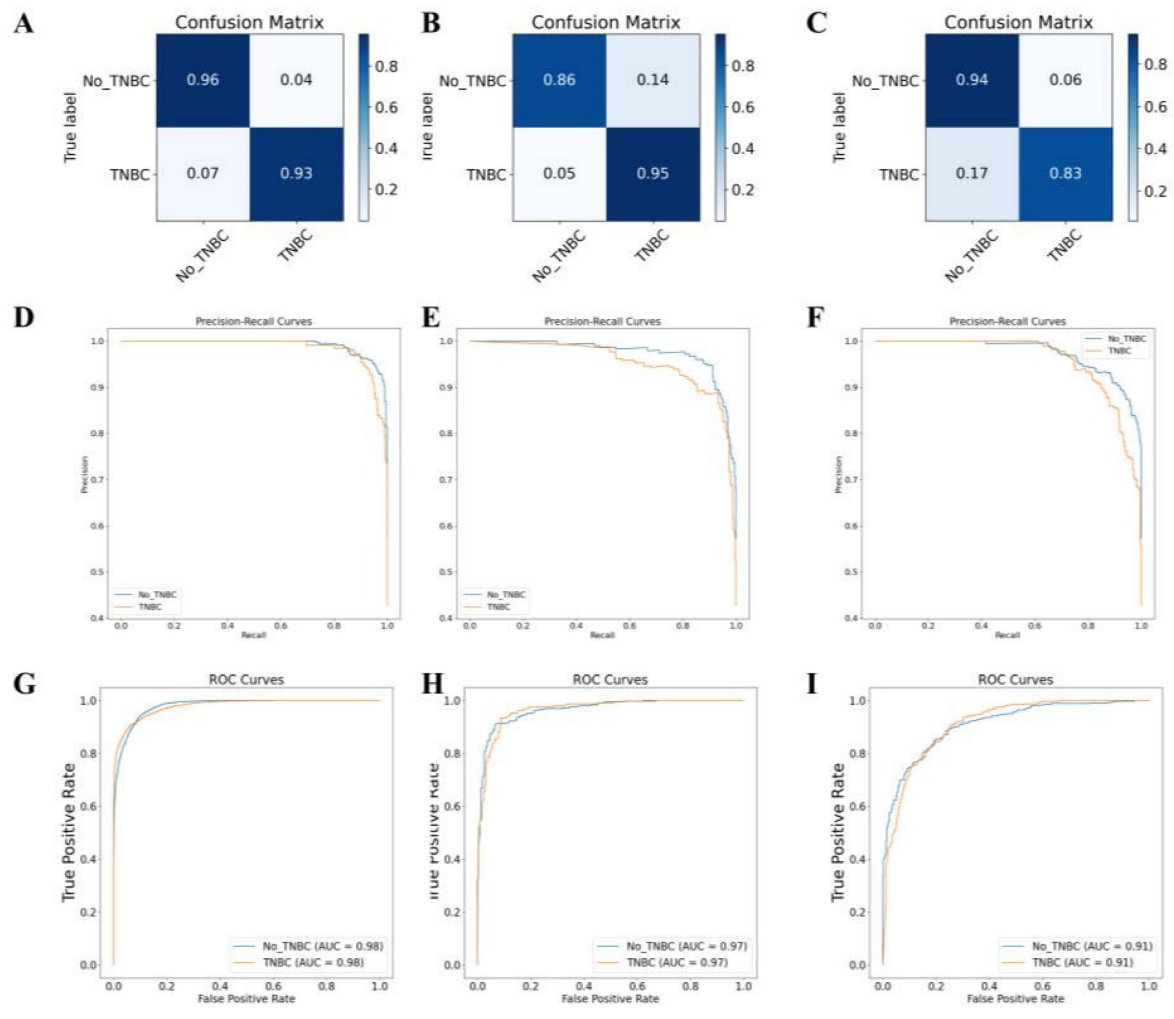
** Gene multi-omics DL model for TNBC subtyping prediction.** Note: (A) Confusion matrix of the gene multi-omics DL model on the training set. (B) Confusion matrix of the gene multi-omics DL model on the validation set. (C) Confusion matrix of the gene multi-omics DL model on the transfer test set. (D) AUC-RF curve of the classification on the training set. (E) AUC-RF curve of the classification on the validation set. (F) AUC-RF curve of the classification on the transfer test set. (G) AUC-ROC curve of the classification on the training set. (H) AUC-ROC curve of the classification on the validation set. (I) AUC-ROC curve of the classification on the transfer test set.

On the TCGA-BRCA dataset, based on the feature scores, this multi-omics training model classified samples into TNBC and non-TNBC. The model achieved prediction accuracies of 98.0%, 97.0%, and 91.0% on the training set, validation set, and external testing set, respectively (Figure 7A-F). The classification results of the model are shown in Table S1. The accuracy, precision, recall, and F1 score values indicate that the multi-omics DL model has high accuracy in identifying the TNBC regular samples. The high AUC values of the training and validation set ROC curves indicate that the model successfully avoided overfitting of the neural network model. Additionally, the AUC-ROC values for the binary classification task exceeded 0.91, indicating that the model effectively distinguishes between the two sample categories (Figure 7G-I).

### Predictive classification and performance evaluation of TNBC using MRI image DL models

In this study, we utilized an MRI image DL model for the predictive classification of TNBC. These images underwent independent blind review by two experienced breast radiologists to ensure objectivity in the analysis. By employing precise image processing techniques, we accurately determined the location of the primary tumor and performed tumor segmentation in the MRI images using the calculated fuzzy mean algorithm. Subsequently, we conducted quantitative radiomics analysis to extract 38 radiomic features, encompassing various aspects such as tumor size, shape, morphology, and texture, which were categorized into four MRI phenotypic types: size, shape, morphology, and texture.

**Figure 8:**
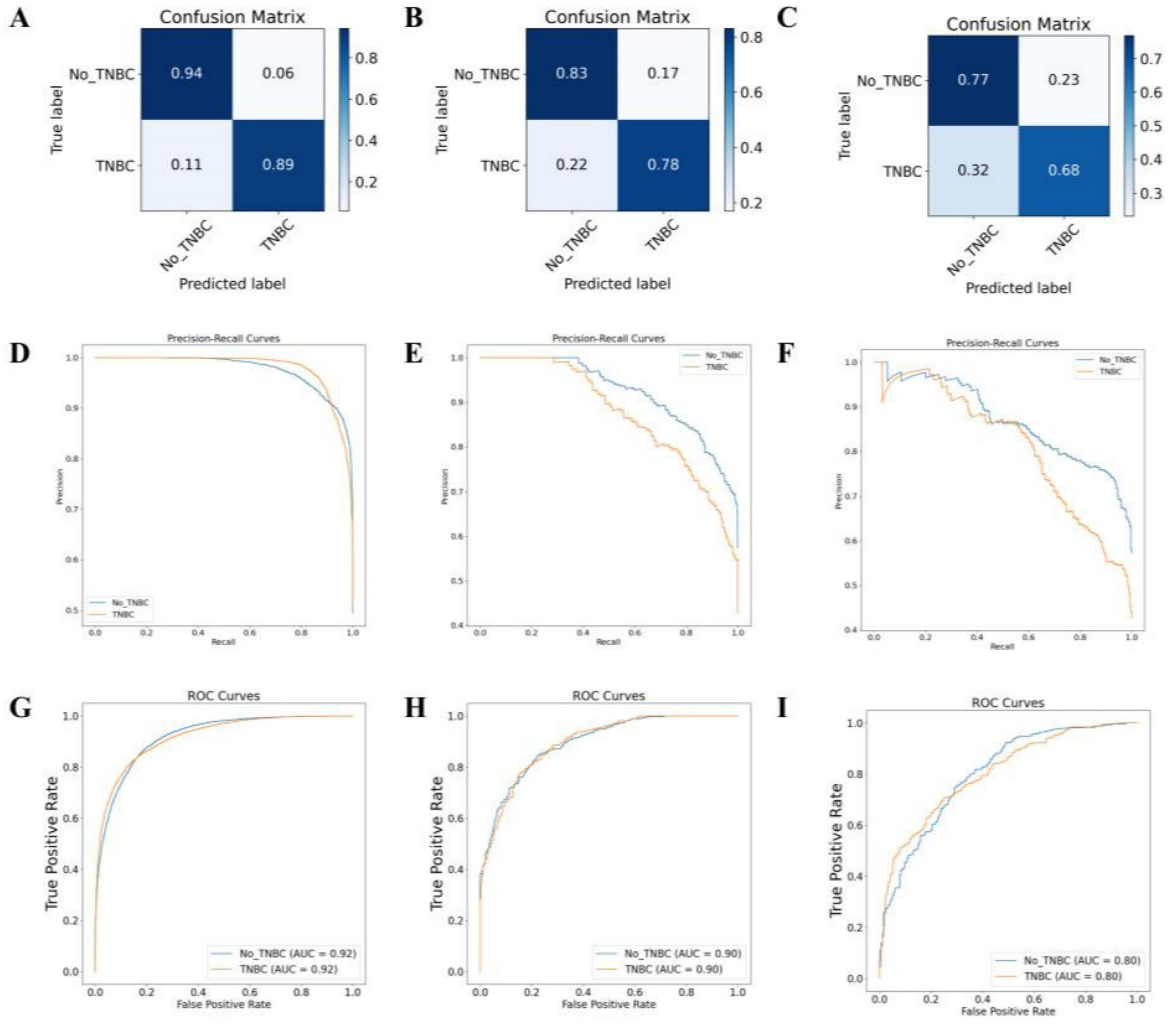
** MRI-based DL model for TNBC subtyping prediction performance.** Note: Confusion matrices of the MRI image DL model on the training set (A), validation set (B), and transfer test set (C); AUC-RF curves of the classification on the training set (D), validation set (E), and transfer test set (F); AUC-ROC curves of the classification on the training set (G), validation set (H), and transfer test set (I).

Following the training and validation of the dataset based on these radiographic features, transfer testing was performed on the TCIA-BRAC dataset. The model's performance was evaluated using metrics, such as accuracy, precision, recall, F1 score, and AUC (Table S2). Results for the training set are displayed in Figures 8A, 8D, and 8G, while the validation set results are presented in Figures 8B, 8E, and 8H. Additionally, Figures 8C, 8F, and 8I illustrate the results of the transfer testing set. Through analysis of the confusion matrix, it was observed that the MRI-based DL algorithm achieved an accuracy of 89% on the training set samples for TNBC diagnosis, 78% on the validation set samples, and 68% on the transfer testing set samples. The AUC ROC results for binary classification demonstrated that the MRI-based DL algorithm achieved classification accuracies of 92% on the training set samples, 90% on the validation set samples, and 80% on the transfer testing set samples.

Furthermore, we conducted a detailed investigation of representative MRI images in which the model made misclassifications in the validation and testing sets (Figure S6A-H). It was observed that false-positive samples tended to have larger tumor sizes and irregular shapes, while false-negative samples often had smaller tumor sizes and exhibited overlapping shapes and tumor edge diffusion characteristics with normal tissue on vascular imaging. This finding suggests that although DL models have demonstrated strong predictive potential, it is still necessary to refine feature extraction further to improve model performance, particularly in comprehensive training set fitting and enhancing diagnostic accuracy for TNBC.

### Comparative evaluation of TNBC subtyping predictive performance based on ROC curve analysis

In our study, we evaluated the performance of a single genomics machine learning model and two DL models in predicting subtypes of TNBC by comparing the ROC curves (Table S3). Based on the AUC values, the DL models for multi-omics data outperformed the single-genomics machine learning model in terms of predictive performance. Furthermore, the MRI radiomics DL model demonstrated superior accuracy to the single genomics machine learning model, and its accuracy was comparable to that of the multi-gene model. However, the multi-gene model exhibited higher accuracy in transfer testing than the MRI radiomics model. This difference may be attributed to the higher level of digitization and refinement of multi-gene features, while the MRI radiomics features are limited by errors in feature extraction and data processing, especially during cross-dataset transfer where they can be influenced by equipment and contrast agents, leading to biases.

## Discussion

The present study assessed the predictive capabilities of various machine learning and DL models on the subtypes of TNBC, a critical endeavor for tailoring personalized treatment strategies 32, 33. Considering molecular features such as mRNA, miRNA, gene mutations, and DNA methylation, alongside MRI imaging data from the TCGA and TCIA databases, we developed and compared multiple predictive models. This approach aimed to construct and optimize distinct models for single-omics machine learning, DL-based multi-omics data integration, and MRI radiomics DL, employing Bayesian optimization for neural network structure refinement. Model performance was rigorously evaluated through cross-validation and transfer testing methodologies 34-36.

Our findings underscored the notable advantage of the DL model for multi-omics data in accurately predicting TNBC subtypes over models relying solely on single-omics data. This superiority suggests that the integration of diverse molecular features significantly improves prediction accuracy. Moreover, while the MRI radiomics DL model showed promising performance, a decline in efficacy during transfer testing highlighted potential issues related to data consistency and calibration within MRI datasets. Contrasting with previous research predominantly focused on single-omics data and conventional machine learning techniques 37, our study is distinguished by its comprehensive use of multi-omics data, including MRI radiomics, analyzed through advanced DL models 38-40. This methodological advancement facilitates more complex and precise predictions of TNBC subtyping subtypes 41. We conducted a comprehensive evaluation of model accuracy, precision, recall, F1 score, and AUC value to demonstrate the effectiveness of the models in distinguishing between triple-negative and non-TNBC. These metrics, widely recognized for evaluating predictive model performance, confirmed the robustness of our models in distinguishing between TNBC and non-TNBC cases. Our models demonstrated exemplary performance across these parameters, indicating their potential to significantly contribute to the field of TNBC subtyping diagnosis and treatment 42.

This study offers novel insights into enhancing diagnostic accuracy and personalizing treatment by integrating multi-omics data with DL technologies. Specifically, the high performance of the DL model for multi-omics data is attributed to its capacity to aggregate and analyze extensive biomolecular data 43, while the MRI radiomics model provides a non-invasive diagnostic approach 44. The synergy of these models holds the potential to refine the classification of TNBC, thereby enabling the formulation of more precise treatment plans for patients 45.

The findings of this research can serve as a valuable tool to assist clinical decision-making. By predicting subtype outcomes, physicians can make more informed judgments about a patient's potential response to certain treatments, optimizing therapeutic strategies accordingly. Furthermore, these models have the potential to be integrated with other biomarkers or clinical parameters in the future, enhancing the accuracy and reliability of predictions. Accurate subtype prediction not only aids physicians in planning treatment but also empowers patients with more information about their disease state and treatment options 46. This increase in information transparency and patient involvement is likely to improve treatment adherence and satisfaction among patients 47. Our study also unveils the potential application of DL in medical diagnostics, indicating a promising direction for future research in medical technology and clinical practice.

However, our study has several limitations. Firstly, despite analyzing a large volume of data, biases and restrictions inherent to our data sources persist. This condition suggests that our findings may be influenced by specific attributes of these sources, which may not be fully applicable across all patient groups. Secondly, our research involves the integration and analysis of multi-omics data, a process dependent on complex algorithms and workflows. Another aspect not adequately addressed in our study is the lack of original images from the DL-based multi-omics data integration approaches due to the vast amount of data generated by the DL models and an initial oversight in preserving complete raw analytical data. The findings were presented through charts and graphs to convey the experimental results clearly. The multi-gene deep learning approach and its results have shown potential clinical application in TNBC diagnosis and prognosis prediction. The high accuracy, precision, recall, and excellent AUC performance indices of our model indicate prospects for aiding diagnostic and personalized treatment decisions.

Future studies are needed to collect and publicly share original image data for detailed evaluation. While these methods demonstrate potential advantages, they still require further optimization and detailed validation to ensure their applicability and stability in various contexts. Additionally, while the DL models showcased encouraging performance in our study, their inherent "black box" nature poses challenges to the interpretability and transparency of the models. This condition is particularly critical in the clinical decision-making process, where physicians and patients often need to understand the logic behind decisions. Thus, further application of DL models should concentrate on improving their interpretability and validating their clinical applicability.

Another limitation of our study was the inability to include an external validation set to corroborate our findings further. This limitation is ascribed to significant challenges in acquiring suitable independent clinical sample sets, particularly considering data accessibility and privacy issues. Therefore, although our models demonstrated good performance on internal datasets, their universal applicability in clinical practice requires cautious evaluation.

Future research could be expanded in scale and sample size to enhance the models' stability and generalizability. Collaboration with other clinical studies and databases could help further validate the models' effectiveness and reliability. Moreover, integrating multi-omics data analysis with other bioinformatics data could deepen our understanding of carcinogenic mechanisms of TNBC subtyping. Lastly, further investigation into the interpretability and explainability of DL models could improve their reliability and acceptability in clinical practice.

In summary, the scientific and clinical value of our study lies in exploring the application of integrating multi-omics data with DL models in TNBC subtyping subtype prediction, offering new methods and insights for personalized treatment and prognosis assessment of TNBC subtyping. Despite some limitations, advancements in future research will continue to propel TNBC subtyping research and clinical practice forward.

## Supplementary Material

Supplementary figures.

## Data Availability

All data can be provided as needed.

## References

[1] Makino Y, Kawamata A, Hikino H, Murata Y, Takahashi T, Miura H (2020). [A Case of Primary Acinic Cell Carcinoma of the Breast]. Gan To Kagaku Ryoho.

[2] Howard FM, Olopade OI (2021). Epidemiology of Triple-Negative Breast Cancer: A Review. Cancer J.

[3] Won KA, Spruck C (2020). Triple-negative breast cancer therapy: Current and future perspectives (Review). Int J Oncol.

[4] Hong R, Xu B (2022). Breast cancer: an up-to-date review and future perspectives. Cancer Commun (Lond).

[5] Zou Y, Xie J, Zheng S, Liu W, Tang Y, Tian W (2022). Leveraging diverse cell-death patterns to predict the prognosis and drug sensitivity of triple-negative breast cancer patients after surgery. Int J Surg.

[6] Gong Y, Ji P, Yang YS, Xie S, Yu TJ, Xiao Y (2021). Metabolic-Pathway-Based Subtyping of Triple-Negative Breast Cancer Reveals Potential Therapeutic Targets. Cell Metab.

[7] Wolf DM, Yau C, Wulfkuhle J, Brown-Swigart L, Gallagher RI, Lee PRE (2022). Redefining breast cancer subtypes to guide treatment prioritization and maximize response: Predictive biomarkers across 10 cancer therapies. Cancer Cell.

[8] Sharma H, Ruikar M (2022). Kangaroo mother care (KMC) for procedural pain in infants: A meta-analysis from the current evidence of randomized control trials and cross-over trials. J Family Med Prim Care.

[9] Hong J, Boussetta N, Enderlin G, Merlier F, Grimi N (2022). Degradation of Residual Herbicide Atrazine in Agri-Food and Washing Water. Foods.

[10] Zarkami R, Abedini A, Sadeghi Pasvisheh R (2022). Analysis of the eutrophication in a wetland using a data-driven model. Environ Monit Assess.

[11] Yin L, Duan JJ, Bian XW, Yu SC (2020). Triple-negative breast cancer molecular subtyping and treatment progress. Breast Cancer Res.

[12] Reddy TP, Rosato RR, Li X, Moulder S, Piwnica-Worms H, Chang JC (2020). A comprehensive overview of metaplastic breast cancer: clinical features and molecular aberrations. Breast Cancer Res.

[13] Onkar SS, Carleton NM, Lucas PC, Bruno TC, Lee AV, Vignali DAA (2023). The Great Immune Escape: Understanding the Divergent Immune Response in Breast Cancer Subtypes. Cancer Discov.

[14] Fang S, Chen B, Zhang Y, Sun H, Liu L, Liu S (2023). Computational Approaches and Challenges in Spatial Transcriptomics. Genomics Proteomics Bioinformatics.

[15] Din NMU, Dar RA, Rasool M, Assad A (2022). Breast cancer detection using deep learning: Datasets, methods, and challenges ahead. Comput Biol Med.

[16] Balkenende L, Teuwen J, Mann RM (2022). Application of Deep Learning in Breast Cancer Imaging. Semin Nucl Med.

[17] Lotter W, Diab AR, Haslam B, Kim JG, Grisot G, Wu E (2021). Robust breast cancer detection in mammography and digital breast tomosynthesis using an annotation-efficient deep learning approach. Nat Med.

[18] Cao LL, Peng M, Xie X, Chen GQ, Huang SY, Wang JY (2022). Artificial intelligence in liver ultrasound. World J Gastroenterol.

[19] Egger J, Gsaxner C, Pepe A, Pomykala KL, Jonske F, Kurz M (2022). Medical deep learning-A systematic meta-review. Comput Methods Programs Biomed.

[20] Majumder A, Sen D (2021). Artificial intelligence in cancer diagnostics and therapy: current perspectives. Indian J Cancer.

[21] Yang X, Zheng Y, Xing X, Sui X, Jia W, Pan H (2022). Immune subtype identification and multi-layer perceptron classifier construction for breast cancer. Front Oncol.

[22] Timmons JA, Anighoro A, Brogan RJ, Stahl J, Wahlestedt C, Farquhar DG (2022). A human-based multi-gene signature enables quantitative drug repurposing for metabolic disease. Elife.

[23] Chi M, Liu J, Mei C, Shi Y, Liu N, Jiang X (2022). TEAD4 functions as a prognostic biomarker and triggers EMT via PI3K/AKT pathway in bladder cancer. J Exp Clin Cancer Res.

[24] Bernal Rubio YL, Gonzalez-Reymundez A, Wu KH, Griguer CE, Steibel JP, de Los Campos G (2018). Whole-Genome Multi-omic Study of Survival in Patients with Glioblastoma Multiforme. G3 (Bethesda).

[25] Saha A, Harowicz MR, Grimm LJ, Kim CE, Ghate SV, Walsh R (2018). A machine learning approach to radiogenomics of breast cancer: a study of 922 subjects and 529 DCE-MRI features. Br J Cancer.

[26] Guo YY, Huang YH, Wang Y, Huang J, Lai QQ, Li YZ (2022). Breast MRI Tumor Automatic Segmentation and Triple-Negative Breast Cancer Discrimination Algorithm Based on Deep Learning. Comput Math Methods Med.

[27] Boulenger A, Luo Y, Zhang C, Zhao C, Gao Y, Xiao M (2023). Deep learning-based system for automatic prediction of triple-negative breast cancer from ultrasound images. Med Biol Eng Comput.

[28] Alghamedy FH, Shafiq M, Liu L, Yasin A, Khan RA, Mohammed HS (2022). Machine Learning-Based Multimodel Computing for Medical Imaging for Classification and Detection of Alzheimer Disease. Comput Intell Neurosci.

[29] Felfeliyan B, Hareendranathan A, Kuntze G, Jaremko JL, Ronsky JL (2022). Improved-Mask R-CNN: Towards an accurate generic MSK MRI instance segmentation platform (data from the Osteoarthritis Initiative). Comput Med Imaging Graph.

[30] Fan M, Xia P, Liu B, Zhang L, Wang Y, Gao X (2019). Tumour heterogeneity revealed by unsupervised decomposition of dynamic contrast-enhanced magnetic resonance imaging is associated with underlying gene expression patterns and poor survival in breast cancer patients. Breast Cancer Res.

[31] Malik V, Kalakoti Y, Sundar D (2021). Deep learning assisted multi-omics integration for survival and drug-response prediction in breast cancer. BMC Genomics.

[32] Zhou BY, Wang LF, Yin HH, Wu TF, Ren TT, Peng C (2021). Decoding the molecular subtypes of breast cancer seen on multimodal ultrasound images using an assembled convolutional neural network model: A prospective and multicentre study. EBioMedicine.

[33] Inglese M, Ferrante M, Boccato T, Conti A, Pistolese CA, Buonomo OC (2023). Dynomics: A Novel and Promising Approach for Improved Breast Cancer Prognosis Prediction. J Pers Med.

[34] Ye Z, Zhang H, Kong F, Lan J, Yi S, Jia W (2021). Comprehensive Analysis of Alteration Landscape and Its Clinical Significance of Mitochondrial Energy Metabolism Pathway-Related Genes in Lung Cancers. Oxid Med Cell Longev.

[35] Ghosh S, Bhowmik S, Majumdar S, Goswami A, Chakraborty J, Gupta S (2020). The exosome encapsulated microRNAs as circulating diagnostic marker for hepatocellular carcinoma with low alpha-fetoprotein. Int J Cancer.

[36] Ye H, Li T, Wang H, Wu J, Yi C, Shi J (2021). TSPAN1, TMPRSS4, SDR16C5, and CTSE as Novel Panel for Pancreatic Cancer: A Bioinformatics Analysis and Experiments Validation. Front Immunol.

[37] Nyamundanda G, Eason K, Guinney J, Lord CJ, Sadanandam A (2020). A Machine-Learning Tool Concurrently Models Single Omics and Phenome Data for Functional Subtyping and Personalized Cancer Medicine. Cancers (Basel).

[38] Wen X, Leng P, Wang J, Yang G, Zu R, Jia X (2022). Clinlabomics: leveraging clinical laboratory data by data mining strategies. BMC Bioinformatics.

[39] Erfanian N, Heydari AA, Feriz AM, Ianez P, Derakhshani A, Ghasemigol M (2023). Deep learning applications in single-cell genomics and transcriptomics data analysis. Biomed Pharmacother.

[40] Wang K, Abid MA, Rasheed A, Crossa J, Hearne S, Li H (2023). DNNGP, a deep neural network-based method for genomic prediction using multi-omics data in plants. Mol Plant.

[41] Zhang X, Zhang Y, Zhang G, Qiu X, Tan W, Yin X (2022). Deep Learning With Radiomics for Disease Diagnosis and Treatment: Challenges and Potential. Front Oncol.

[42] Velasquez-Martinez L, Caicedo-Acosta J, Acosta-Medina C, Alvarez-Meza A, Castellanos-Dominguez G (2020). Regression Networks for Neurophysiological Indicator Evaluation in Practicing Motor Imagery Tasks. Brain Sci.

[43] Poirion OB, Jing Z, Chaudhary K, Huang S, Garmire LX (2021). DeepProg: an ensemble of deep-learning and machine-learning models for prognosis prediction using multi-omics data. Genome Med.

[44] Conti A, Duggento A, Indovina I, Guerrisi M, Toschi N (2021). Radiomics in breast cancer classification and prediction. Semin Cancer Biol.

[45] Chaddad A, Tan G, Liang X, Hassan L, Rathore S, Desrosiers C (2023). Advancements in MRI-Based Radiomics and Artificial Intelligence for Prostate Cancer: A Comprehensive Review and Future Prospects. Cancers (Basel).

[46] Olbrecht S, Busschaert P, Qian J, Vanderstichele A, Loverix L, Van Gorp T (2021). High-grade serous tubo-ovarian cancer refined with single-cell RNA sequencing: specific cell subtypes influence survival and determine molecular subtype classification. Genome Med.

[47] Marisa L, de Reynies A, Duval A, Selves J, Gaub MP, Vescovo L (2013). Gene expression classification of colon cancer into molecular subtypes: characterization, validation, and prognostic value. PLoS Med.

