# Supplementary material for: Comparative Evaluation of Machine Learning Models for Subtyping Triple-Negative Breast Cancer: A Deep Learning-Based Multi-Omics Data Integration Approach
Source: J Cancer. 2024 May 30;15(12):3943–57. doi: 10.7150/jca.93215 (PMC11190774; doi:10.7150/jca.93215)
Supplement: Supplementary file 1 — Supplementary figures. [file jcav15p3943s1.pdf]

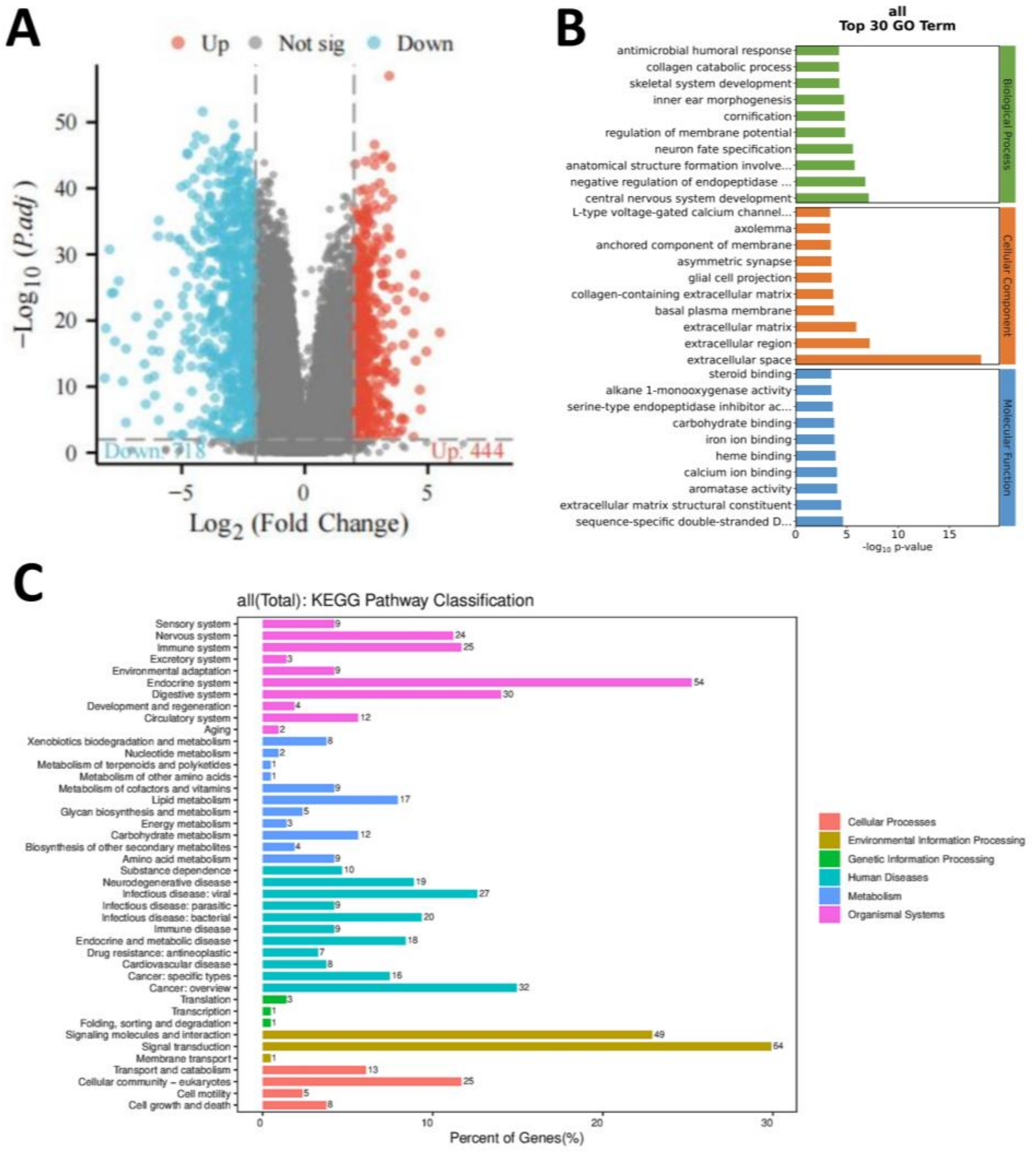

Figure S1. Differential expression of TNBC-positive and -negative samples at the transcriptome level.

Note: (A) Volcano plot showing the differential RNA expression between TNBC-positive and -negative samples. Red represents significantly downregulated TNBC-positive samples, blue represents significantly upregulated TNBC-negative samples, and gray represents samples with no significant difference. (B) GO enrichment analysis of DEGs in TNBC-positive samples, with the size

of the points indicating the number of genes in the pathway and the color indicating the adjusted P-value. (C) KEGG enrichment analysis of DEGs in TNBC-positive samples, with the size of the points indicating the number of genes in the pathway and the color indicating the adjusted P-value.

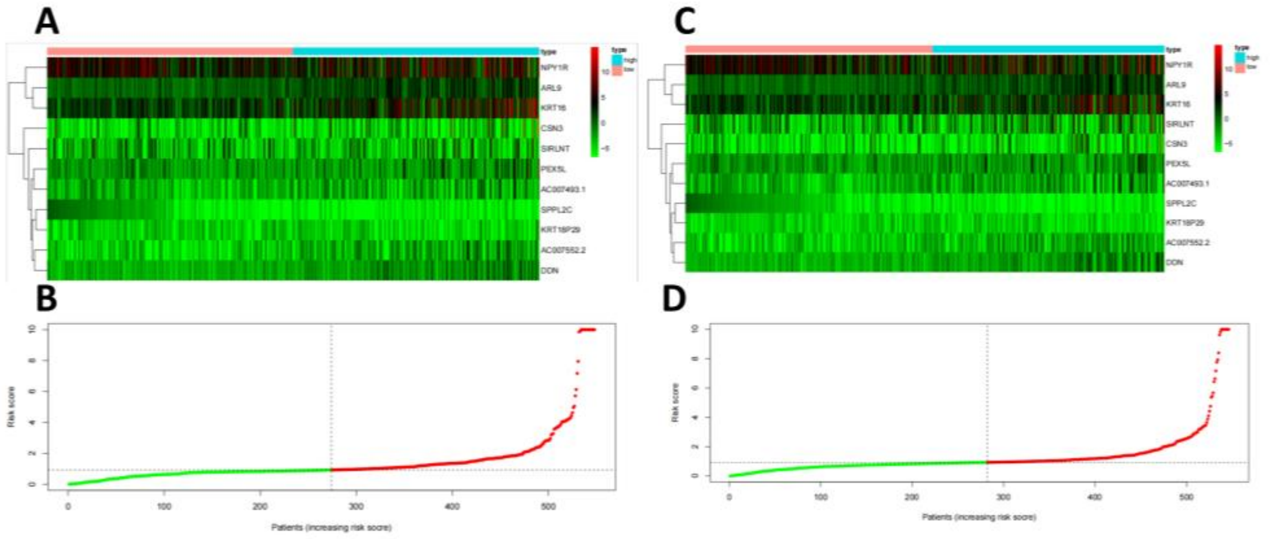

Figure S2. Gene heatmap and risk curve of miRNA-seq data in the training and validation sets. Note: (A) Heatmap of DEGs between high-risk and low-risk groups in the training set. (B) The risk curve of the training set, where the X-axis represents patients sorted by increasing risk score and the Y-axis represents the computed risk score. The green region represents the low-risk group, and the red region represents the high-risk group. (C) Heatmap of DEGs between high-risk and low-risk groups in the validation set. (D) Risk curve of the validation set, with the X-axis representing patients sorted by increasing risk score and the Y-axis representing the computed risk score. The green region represents the low-risk group, and the red region represents the high-risk group.

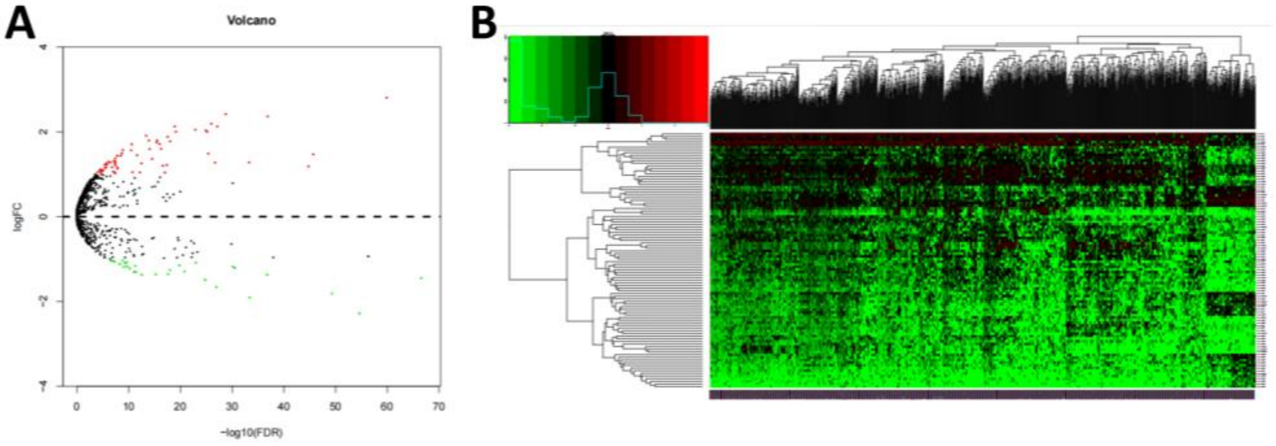

Figure S3. Differences in transcriptomes between TNBC-positive and -negative samples.

Note: (A) Volcano plot showing differential miRNA expression between TNBC-positive and -negative samples. Red indicates significantly downregulated expression in TNBC-positive samples, blue indicates significantly upregulated expression, and black indicates no significant difference. (B) Heatmap of miRNA expression differences between TNBC-positive and -negative samples.

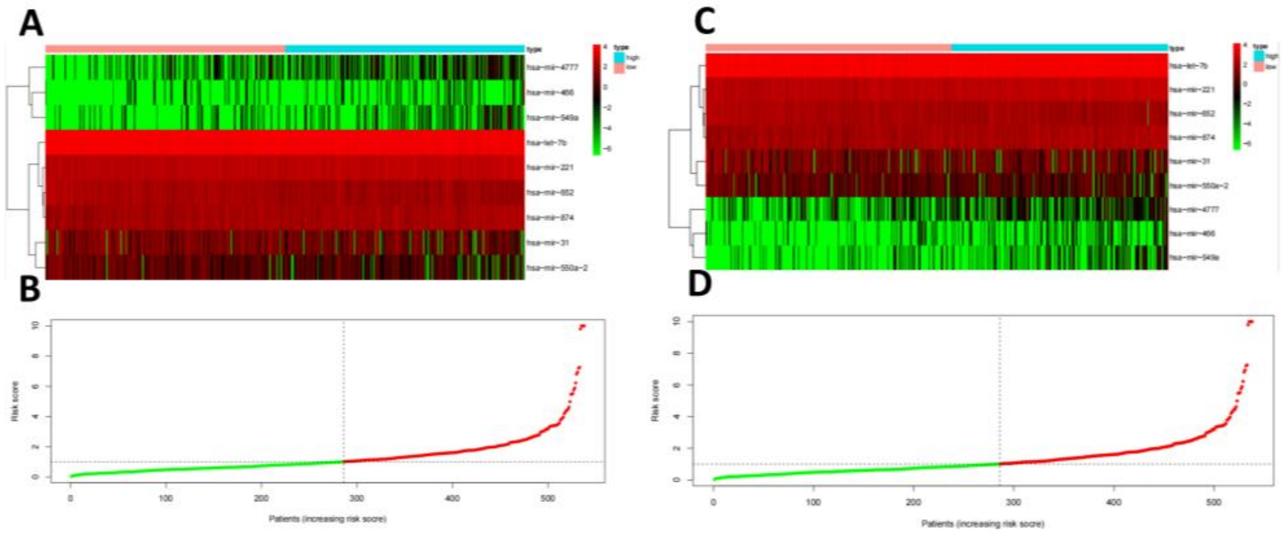

Figure S4. Heatmap and risk curve of miRNA-seq data training and validation sets.

Note: (A) Heatmap showing differential gene expression between high-risk and low-risk groups in the training set of the model. (B) Risk curve of the training set, with patients sorted by risk score on the x-axis and computed risk scores on the y-axis. The green region represents the low-risk group, and the red region represents the high-risk group. (C) Heatmap showing differential gene expression between high-risk and low-risk groups in the validation set of the model. (D) Risk curve of the validation set, with patients sorted by risk score on the x-axis and computed risk scores on the y-axis. The green region represents the low-risk group, and the red region represents the high-risk group.

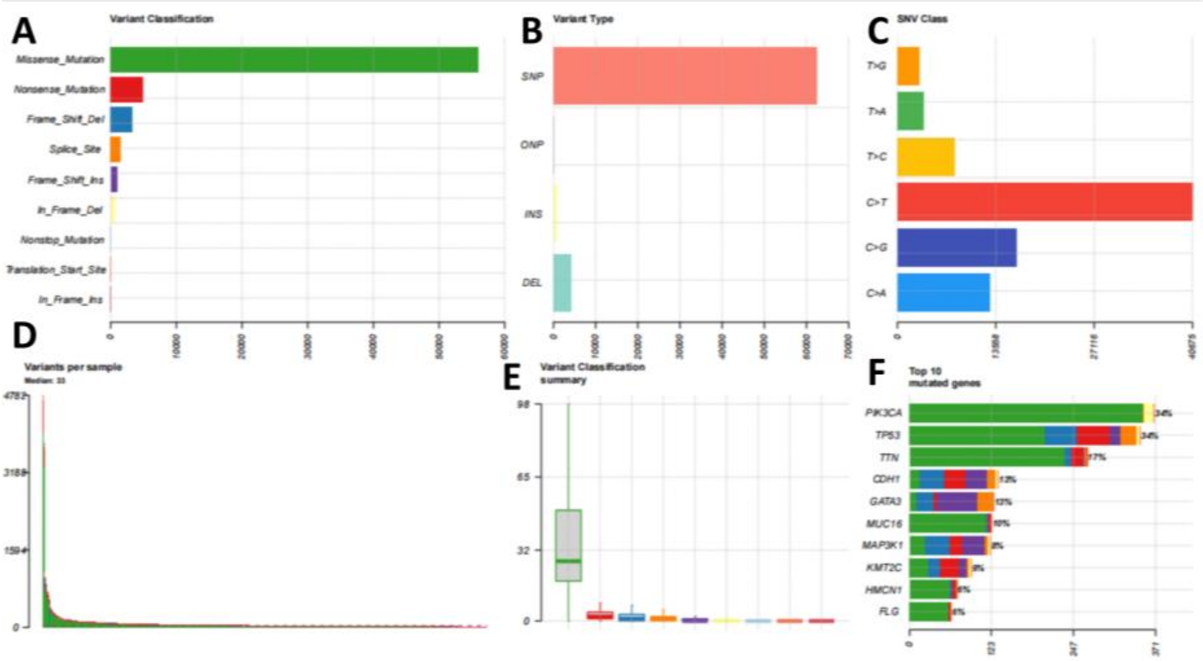

Figure S5. Gene mutation features in TNBC-positive and -negative samples.

Note: (A) Variant Classification: types of mutations. (B) Variant Type: Primarily single nucleotide polymorphisms (SNP), which are significantly outnumbering other types. (C) SNV Class: classification of single nucleotide variations (SNVs). (D) Variants per Sample: distribution of the number of variants per sample. (E) Variant Classification Summary:distribution and median of different variant classifications in the form of a boxplot. (F) Top 10 Mutated Genes: top 10 genes with the largest number of mutations.

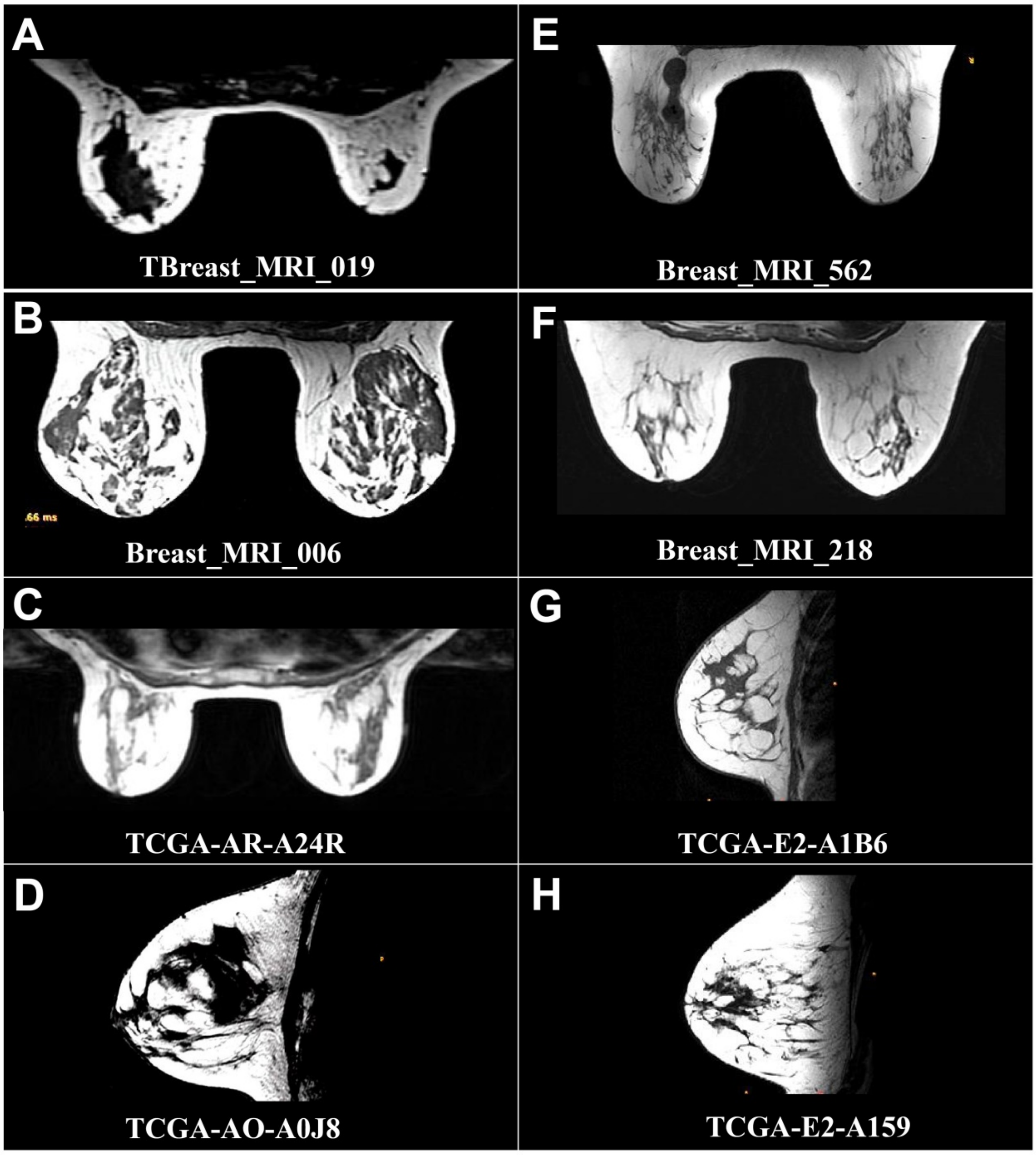
A
TBreast_MRI_019
E
Breast_MRI_562
B
Breast_MRI_006
F
Breast_MRI_218
C
TCGA-AR-A24R
G
TCGA-E2-A1B6
D
TCGA-AO-A0J8
H
TCGA-E2-A159

Figure S6. Representative misclassified images under the DL model.

Note: (A-D) False positive samples: Non-TNBC breast samples misclassified as TNBC. (E-H) False negative samples: TNBC samples misclassified as non-TNBC. The numbers below the images indicate the sample identification in the original dataset.
